# Pocket Infection Leading to Complete Extrusion of an Implantable Cardiac Defibrillator

**DOI:** 10.7759/cureus.90392

**Published:** 2025-08-18

**Authors:** Wassim Beladel, Soukaina Aabbar, Karim Hasni, Mohamed El Minaoui

**Affiliations:** 1 Cardiology Department, University Hospital Souss Massa, Agadir Faculty of Medicine and Pharmacy, Ibn Zohr University, Agadir, MAR; 2 Cardiology Department, Toulon Hospital Center, Toulon, FRA

**Keywords:** device complete extrusion, implantable defibrillator, infection, infective endocarditis, pocket infection

## Abstract

Pocket infection and complete device extrusion are uncommon but potentially life-threatening complications that present significant management challenges. Extrusion results from progressive skin erosion until the device exits the subcutaneous pocket, at which point the risk of bacterial contamination becomes high. Diagnosis of extrusion is based primarily on clinical examination, while the diagnosis of cardiac implantable electronic device (CIED) endocarditis relies on the modified Duke criteria in conjunction with multimodality imaging. Alongside echocardiography, 18F-FDG PET-CT is increasingly used for the diagnosis of endocarditis; it is particularly valuable when the diagnosis is uncertain and contributes to identifying potential portals of entry. Management depends on the extent of infection and requires bactericidal antibiotic therapy combined with complete device and lead extraction, followed by re-implantation of a new system when clinically appropriate. Early recognition of erosion signs before the device breaches the skin, along with rigorous measures to prevent pocket contamination and subsequent endocarditis, remains crucial to avoid severe complications.

## Introduction

In recent years, there has been a global increase in the number of cardiac implantable electronic devices (CIED). Nowadays, approximately 1.2 million CIED are implanted each year worldwide [1]. This rising pattern stems from broadening indications, encompassing patients who benefit from cardiac resynchronization therapy devices, prophylactic implantable cardioverter defibrillators, and an escalating demand for permanent pacing among an aging patient demographic with associated risk factors. Undesirably, cardiac device-related endocarditis is a rising phenomenon, representing around 5% of all endocarditis; it is a life-threatening complication with challenging management and the primary cause of morbidity and mortality in CIED implantations [1]. Complete CIED extrusion out of the subcutaneous pocket is a rare complication after device implantation and can be due to skin erosion. It is associated with a greater risk of life-threatening complications, especially bacterial contamination.

In this case report, we present a 66-year-old male patient with CIED-related endocarditis due to complete device extrusion and oxacillin-resistant *Staphylococcus epidermidis* (SE).

## Case presentation

A 66-year-old male patient with a medical history of coronary artery disease treated with stenting and implanted seven years earlier with a dual-chamber implantable cardioverter-defibrillator (ICD), underwent device replacement. A device check six weeks after the procedure revealed no evidence of wound dehiscence or local skin infection. Five months later, complete extrusion of the device occurred. The patient reported having noticed generator movement under the skin during the month prior to extrusion, but denied any scratching of the overlying skin.

**Figure 1:**
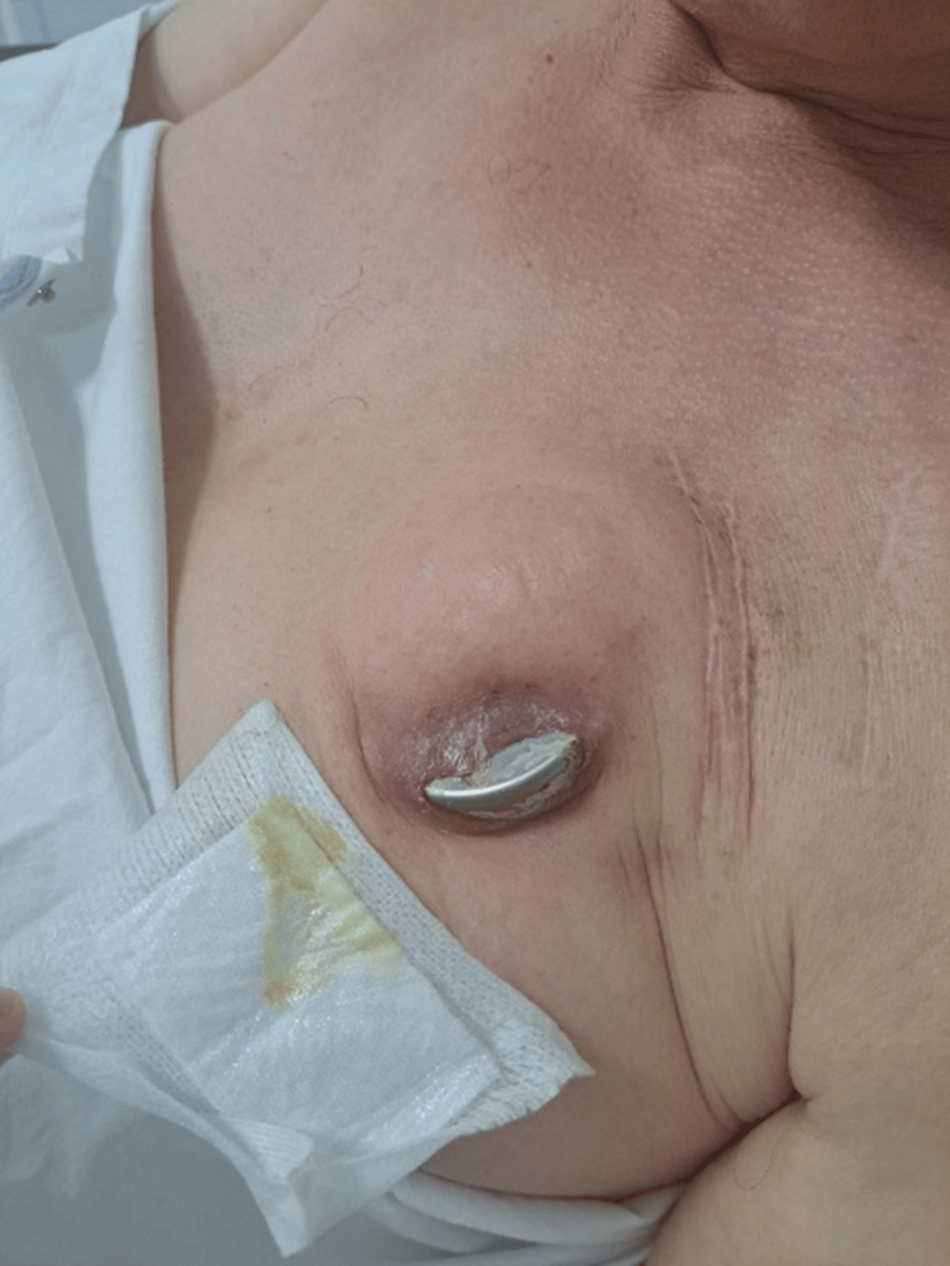
Photograph demonstrating full extrusion of the defibrillator associated with purulent exudate on the wound dressing

**Figure 2:**
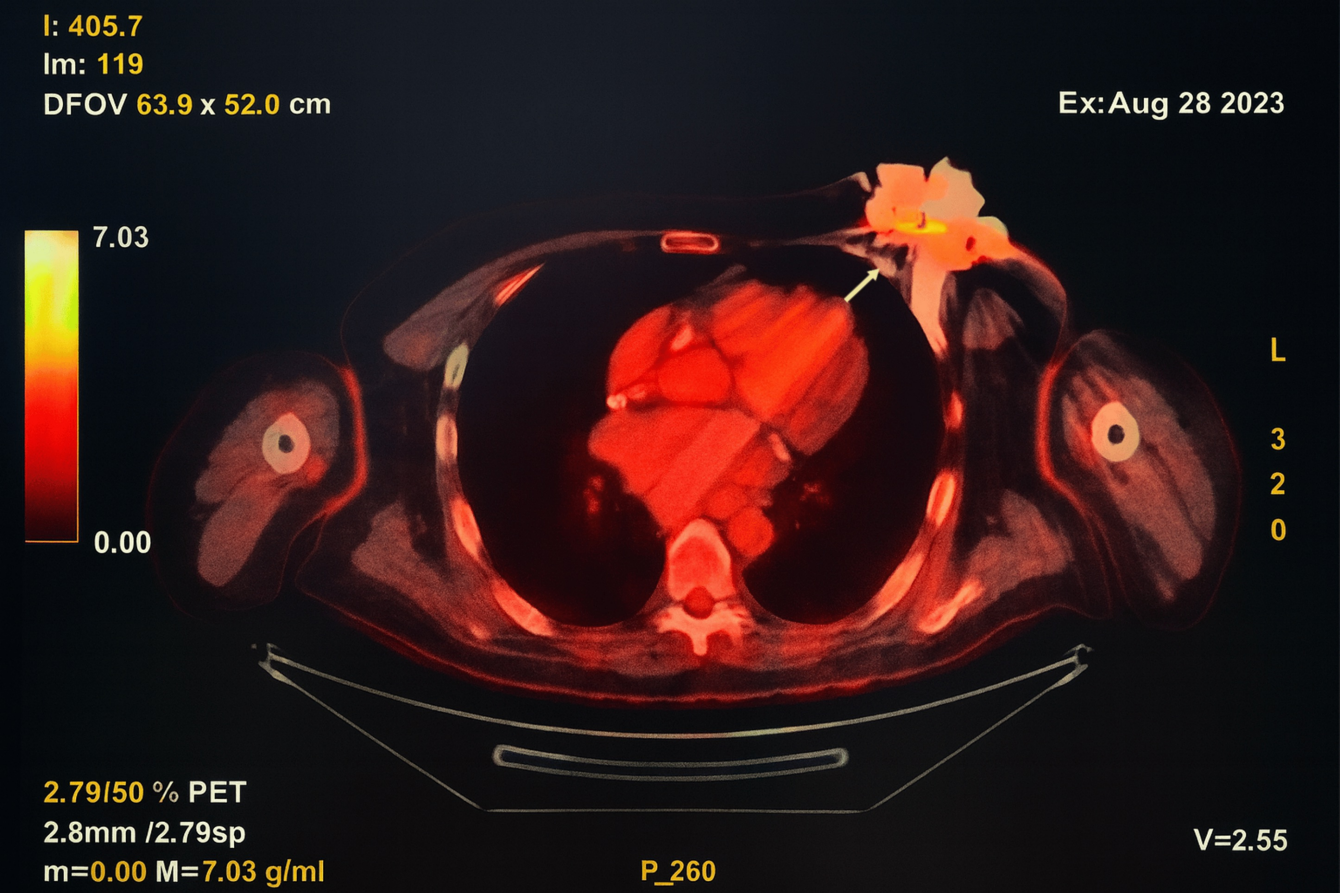
18F-FDG PET-CT showing intense hypermetabolism around the defibrillator

On physical examination, the patient was hemodynamically stable with blood pressure at 120/65 mmHg, heart rate at 88 bpm, and good oxygen saturation and was afebrile. Pocket examination revealed signs of local inflammation with purulent discharge on the wound dressing (Figure 1). The patient did not present systemic inflammatory symptoms or fever. Transthoracic and transesophageal echocardiography showed no evidence suggestive of infectious endocarditis, although three blood cultures were positive for oxacillin-resistant SE. An 18F-FDG PET-CT demonstrated infection of the device casing with intense hypermetabolism around it (Figure 2).

The patient was treated with dual bactericidal antibiotic therapy and underwent complete device extraction. Re-implantation of an ICD was performed on the contralateral side 72 hours after the second negative blood culture, with favorable follow-up.

## Discussion

Infection represents a significant complication of implanted cardiac devices. The misidentification of cardiac device infections is linked to higher mortality rates, while overdiagnosis results in unnecessary device extraction, prolonged hospital stays, and increased mortality rates [2].

Skin erosion by the underlying pacemaker or automated implantable cardioverter-defibrillators is a known CIED complication. It occurs in approximately 0.9% of patients [3]. The complete generator extrusion is a rare late complication. This complication can arise due to a superficial subcutaneous pacemaker pocket or local infection. Some of the risk factors for extrusion are related to compromised immune systems, cognitive impairment in elderly patients, and skin characteristics such as thin subcutaneous fat layers, fragile skin tissue, subsequent skin erosion, poor hygiene, local irritation and infection, and impaired wound healing [4]. The size and configuration of the pocket and the device are additional factors to consider. Device extrusion typically develops after a pre-erosion period characterized by skin changes and tissue thinning. Eventually, the patient may become symptomatic, experiencing discomfort, pain, or pruritus.

The diagnosis of cardiac implantable device endocarditis is established primarily through positive imaging findings in at least 85% of the patients. For many years, echocardiography has been crucial in assessing and treating patients suspected of having infections related to prosthetic devices and CIED. It plays an important role in vegetation and abscesses diagnosis but also assesses functional implications, such as valve regurgitation or stenosis [5,6]. Transesophageal is more sensitive than a transthoracic echocardiogram, although it is not conducted for every patient. Expert guideline documents have designated it as a Class I recommendation for any suspicion of endocarditis [5-7], in case of strong clinical suspicion and a normal echocardiographic examination. In the Modified Duke Criteria, echocardiographic findings indicative of endocarditis are classified as "major" diagnostic criteria for infection [8]. Guideline documents from both US and European societies, along with widely employed clinical diagnostic algorithms, strongly advocate for the performance of a transthoracic echocardiogram in all patients suspected of cardiac device infection. Additional imaging modalities, such as cardiac computed tomography, 18-FDG PET-CT, or leucocyte scintigraphy, are beneficial in instances of challenging diagnosis and are very sensitive exams for the evaluation of prosthetic infective endocarditis and CIED pocket infections. It can either confirm the diagnosis or find evidence of disseminated infection or portals of entry. On CT scans, a generator pocket infection might be identifiable by the presence of fluid surrounding the device with rim enhancement or fat stranding. Nonetheless, metal artifacts caused by the generator significantly limit the effectiveness of CT in detecting pocket infections, making it a secondary diagnostic tool for this purpose. Magnetic resonance imaging can often be performed safely in patients with implanted devices when rigorous protocols are followed. However, it is not recommended for suspected device-related infections since the device itself cannot be visualized due to artifacts caused by the device.

The additional significant criteria in diagnosing endocarditis are a positive blood culture. In the past,* Streptococcus viridans* was frequently isolated as the primary pathogen. However, currently, *Staphylococcus *species are notably more prevalent. However, some studies show that the rate of positive blood cultures in cardiac implantable device endocarditis is notably lower compared to endocarditis not associated with cardiac devices [1].

Furthermore, we should in some cases consider the five minor Modified Duke Criteria, which are predisposing factors such as underlying valvular abnormalities, structural heart disease, or intravenous drug use; fever; presence of vascular manifestations such as mycotic aneurysms, intracranial hemorrhage, Janeway lesions, major arterial emboli, or septic pulmonary infarcts; immunological phenomena such as Osler’s nodes, Roth spots, glomerulonephritis, or positive rheumatoid factor; and positive blood cultures that do not meet the aforementioned major criterion or serological indications of infection consistent with infectious endocarditis [6]. Infections have specific features that render management more challenging, leading to a poorer prognosis.

The treatment of CIED infections requires complete device removal and bactericidal antibiotic therapy. For isolated pocket infections, antibiotics are usually administered for approximately two weeks, whereas, in cases of device-related endocarditis, treatment typically lasts four to six weeks and may be extended up to eight weeks depending on the pathogen and clinical course. As an initial strategy, the probability of staphylococcal involvement often requires starting empirical antibiotic therapy aimed at staphylococci, with subsequent adjustments based on blood culture data. The second fundamental aspect of therapy is the complete removal of the device, followed by re-implantation. Re-implantation is usually performed on the contralateral side; however, if the infection is strictly confined to the pocket and adequately controlled, re-implantation on the same side may be considered [9]. Several solutions for the ablation are technically viable: the transvenous route involves manual traction using non-locking stylets for recent infections, Cook equipment with locking stylets, or the Laser system, if stylet traction is insufficient. In cases of persistent fragments or failure via the transvenous route, the femoral approach allows for the use of simple lasso systems or more advanced options such as the "needle's eye snare." Studies suggest a success rate of around 95% for these techniques. Surgical intervention, with or without extracorporeal circulation, is reserved for cases where percutaneous approaches fail or in the presence of large vegetation (≥ 15 or 20 mm).

The growing complexity of handling these endocarditis cases underscores the heightened significance of prevention. The increased infectious risk associated with the number of procedures and manipulations undergone by the patient, including device replacement, electrode repositioning, or resynchronization techniques, implies a crucial role for antibiotic prophylaxis as a preventive measure. It is now well-established that antibiotic prophylaxis is a crucial step in preventing device infections. The use of a LifeVest may be considered. The prognosis depends on the extent of the infection.

## Conclusions

Complete defibrillator extrusion is an uncommon and potentially life-threatening complication, particularly when associated with endocarditis. Early identification of erosion signs before the hardware breaches the skin and prevention of pocket contamination are crucial.

The clinical presentation of CIED endocarditis is highly variable. The Modified Duke Criteria remain the gold standard for confirming the diagnosis, while risk factors for extrusion should also be considered. General antibiotic prophylaxis has proven effective in prevention.
